# The PALB2 DNA-binding domain is an intrinsically disordered recombinase

**DOI:** 10.21203/rs.3.rs-3235465/v1

**Published:** 2023-09-18

**Authors:** Yevhenii Kyriukha, Maxwell B. Watkins, Jennifer M. Redington, Reza Dastvan, Vladimir N. Uversky, Jesse Hopkins, Nicola Pozzi, Sergey Korolev

**Affiliations:** 1 Edward A. Doisy Department of Biochemistry and Molecular Biology, Saint Louis University School of Medicine, St Louis, MO; 2 BioCat, Advanced Photon Source, Argonne National Lab, Argonne, IL; 3 Department of Molecular Medicine and USF Health Byrd Alzheimer’s Research Institute, Morsani College of Medicine, University of South Florida, Tampa, FL.

## Abstract

The Partner and Localizer of BRCA2 (PALB2) tumor suppressor is a scaffold protein that links BRCA1 with BRCA2 to initiate homologous recombination (HR). PALB2 interaction with DNA strongly enhances HR efficiency. The PALB2 DNA-binding domain (PALB2-DBD) supports DNA strand exchange, a complex multistep reaction supported by only a few protein families such as RecA-like recombinases or Rad52. The mechanisms of PALB2 DNA binding and strand exchange are unknown. We performed circular dichroism, electron paramagnetic spectroscopy, and small-angle X-ray scattering analyses and determined that PALB2-DBD is intrinsically disordered, even when bound to DNA. The intrinsically disordered nature of this domain was further supported by bioinformatics analysis. Intrinsically disordered proteins (IDPs) are prevalent in the human proteome and have many important biological functions. The complexity of the strand exchange reaction significantly expands the functional repertoire of IDPs. The results of confocal single-molecule FRET indicated that PALB2-DBD binding leads to oligomerization-dependent DNA compaction. We hypothesize that PALB2-DBD uses a chaperone-like mechanism to aid formation and resolution of complex DNA and RNA multichain intermediates during DNA replication and repair. Since PALB2-DBD alone or within the full-length PALB2 is predicted to have strong liquid-liquid phase separation (LLPS) potential, protein-nucleic acids condensates are likely to play a role in complex functionality of PALB2-DBD. Similar DNA-binding intrinsically disordered regions may represent a novel class of functional domains that evolved to function in eukaryotic nucleic acid metabolism complexes.

## Introduction

Homologous recombination (HR) is essential for the exchange of genetic information between maternal and paternal alleles and proper chromosome segregation during meiosis, for non-mutagenic repair of large chromosome aberrations such as double-stranded (ds) DNA breaks (DSB), for repair of stalled replication forks, and for a plethora of other DNA transactions^1–4^. HR is almost exclusively supported by a single family of RecA-like recombinases, including phage UvsX, bacterial RecA, archaea RadA, and eukaryotic Rad51 and DMC1^5^. The sequences and structures of these RecA-like recombinases are highly conserved, owing to the complexity of a multistep HR reaction involving the formation of an ATP-dependent protein filament on single-stranded (ss) DNA that is capable of searching for a homologous dsDNA and catalyzing strand displacement and strand exchange^3,6–8^. The complexity of this mechanism limits the evolution of more specialized recombinases. However, numerous partner proteins have evolved to regulate organism- and pathway-specific activities of RecA-like recombinases at multiple levels ^9–13^.

RecA-like recombinases recombine long DNA regions with high fidelity but are not efficient for strand exchange between short DNA fragments or between DNA and RNA, including complex multichain transactions during replication fork reversal or transcription/replication collision. Several other protein families are reported to support DNA-pairing and strand exchange, including exonuclease complexes with DNA-annealing proteins such as *E. coli* RecE/RecT, bacteriophage λ Redα/Redβ, and viral UL12/ICP8 complexes^8,14–16^. Another protein capable of strand exchange is the eukaryotic Rad52^17–19^, which is also a major positive regulator of Rad51 in yeast^20,21^. The described proteins form ring-shaped oligomers^22,23^. Rad52 forms an oligomeric toroidal ring that binds ssDNA and dsDNA to promote promiscuous strand exchange^24–26^. Rad52 is more efficient than Rad51 in strand exchange between ssRNA and dsDNA, and is proposed to repair breaks that occur during collision of replication with transcription using newly transcribed mRNA as a homologous template^17,27^. The necessity to process complex multichain DNA and RNA intermediates that are formed in chromatin during all major nucleic acid metabolism reactions is further underscored by limited reports of other proteins with strand annealing and exchange activities. These families include the FANCA protein of the Fanconi anemia (FA) complex, which is critical for DNA interstrand crosslink repair^28^, and the RNA-processing FET proteins such as pro-oncoprotein TLS/FUS and the human splicing factor PSF/hPOMp100^29–31^.

**Fig. 1 |:**
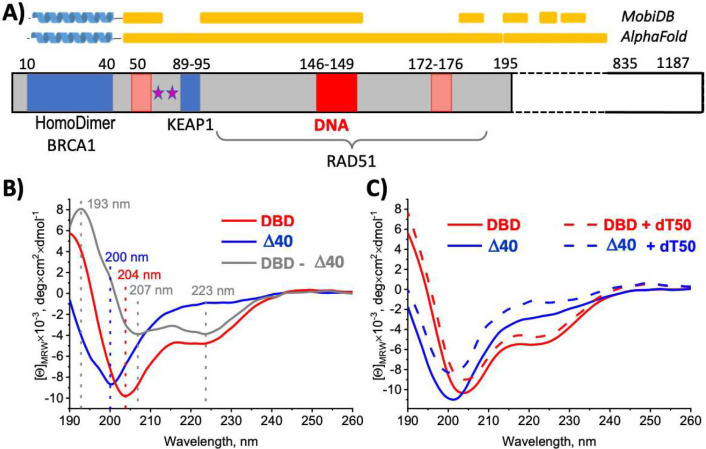
Amino acid sequence features and CD spectroscopy of PALB2-DBD. **(A)** Schematic of PALB2 sequence domains. Amino acid sequence numbers are shown above each domain. Grey area, PALB2-DBD; blue area, known protein interaction sites; red area, major DNA-binding site; pink area, minor DNA-binding sites; stars, phosphorylation sites; yellow bars above, disordered regions according to MobiDB or AlphaFold prediction. **(B)** Circular dichroism (CD) spectra of PALB2-DBD (red), Δ40-DBD (blue), and subtraction of the two spectra (grey). Peaks for each spectrum are marked by dashed lines and numbers of the corresponding color. **(C)** CD spectra of PALB2-DBD (red) and Δ40-DBD (blue) with and without ssDNA shown by dashed and solid lines, respectively.

We discovered that the Partner And Localizer of BRCA2 (PALB2) protein facilitates DNA strand exchange^32^. Human PALB2 is a scaffold protein (1186 amino acids) that links BRCA1 and BRCA2 during HR initiation, where BRCA2 stimulates RAD51 filament formation^33–39^. PALB2 interacts with numerous other chromatin and transcription factors, and with RAD51 and its paralogs^34^. The N-terminal domain encompassing amino acids 1–195 interacts with DNA (referred to hereafter as PALB2-DBD) (Fig. 1A)^40,41^. Alanine substitution of major DNA-binding amino acids 146-RRKK-149 decreases radiation-induced RAD51 foci and HR in cells by 50%^32^. We discovered that PALB2-DBD alone promotes RAD51-independent strand exchange *in vitro*^32^. PALB2 lacks any sequence or predicted structural similarities with Rad51 or Rad52, and likely uses a novel mechanism for strand exchange.

The PALB2 protein has a low complexity amino acid sequence, which is characteristic for scaffold IDPs^42^ (Fig. 1A) (https://MobiDB.bio.unipd.it/Q86YC2 and https://alphafold.ebi.ac.uk/entry/Q86YC2)^43,44^, except for two folded regions that include the N-terminal α-helix (aa 10–40)^45^ and the C-terminal WD40 domain (aa 835–1186)^46^. The N-terminal α-helix forms an antiparallel coiled-coil either with itself (referred to hereafter as PALB2-cc) to form a homodimer, or with the BRCA1 α-helix^47–49^. The remaining PALB2-DBD region (aa 41–195, referred to hereafter as Δ40-DBD) is a neutral peptide with a balanced content of hydrophobic, positively, and negatively charged amino acids characteristic for a context-dependent structural organization^50–54^ (Extended Data Fig. 1). The solution properties, oligomerization, localized DNA-binding site, and complex strand exchange activity of PALB2-DBD are not typical for IDRs, and suggest that oligomerization and/or DNA binding can stimulate the formation of additional α-helixes and PALB2-DBD folding. In line with this hypothesis, multiple segments of this protein (aa 5–46, 49–58, 70–79, 86–100, 118–129, 135–143, 145–167, and 174–194) were identified by FuzDrop (https://fuzdrop.bio.unipd.it/predictor)^55^ as regions with context-dependent interactions (Extended Data Fig. 1F). Furthermore, the PALB2-DBD is predicted to contain six disorder-based protein-protein interaction sites (aa 18–35, 74–86, 98–106, 113–118, 154–174, and 193–206) known as molecular recognition features (MoRFs), which are intrinsically disordered regions (IDRs) undergoing binding-induced folding at interaction with specific partners^56–61^ (Extended Data Fig. 1G).

Here, we present evidence that PALB2-DBD is an intrinsically disordered domain (IDD) or IDR with and without DNA. We characterize its interaction with ssDNA and suggest a novel chaperone-like strand exchange mechanism. PALB2-DBD is the first example of an IDD/IDR that supports a multistep reaction with two complex substrates. This significantly expands the structural and functional repertoire of intrinsically disordered proteins (IDPs) and IDDs/IDRs, which represent a third of the human proteome^42^. Approximately 30% and 50% of protein and RNA chaperones are estimated to form IDRs, and the proposed strand exchange mechanism will have broad implications as only a few have been mechanistically characterized^62–64^. Many eukaryotic DNA repair proteins such as BRCA1 and BRCA2 contain DNA-binding IDRs of unknown function. We hypothesize that such regions represent a novel functional class of DNA repair effectors that can aid transactions between DNA and RNA strands to form or resolve local transient multistrand intermediates that occur near stalled replication forks, DNA breaks and other chromosomal aberrations.

## Results

### PALB2-DBD is an intrinsically disordered region

The Δ40-DBD contains several sequence regions with low α-helical propensity that can form a globular structure on oligomerization or DNA binding. We utilized circular dichroism (CD) spectroscopy to detect the presence of secondary structure elements (Fig. 1B). The PALB2-DBD spectra are characterized by peaks corresponding to α-helical and random coil (RC) structures (Fig. 1B). The spectrum of the Δ40-DBD fragment lacking the N-terminal α-helix does not display a peak corresponding to α-helix, whereas subtraction of PALB2-DBD and Δ40-DBD spectra resulted in a typical spectrum for an α-helix. Therefore, CD spectroscopy did not detect secondary structure elements beyond the N-terminal coiled-coil region in PALB2-DBD. To investigate whether additional secondary structure elements can be stabilized by interactions with DNA, we measured CD spectra from PALB2-DBD and Δ40-DBD in the presence of excess ssDNA (dT_50_) (Fig. 1C). The results showed that neither PLAB2-DBD nor Δ40-DBD spectra were altered by addition of excess ssDNA. These combined results suggest that Δ40-DBD is an intrinsically disordered region (IDR). It interacts with DNA as IDR, despite a localized major DNA-binding site typical for globular proteins.

**Fig. 2 |:**
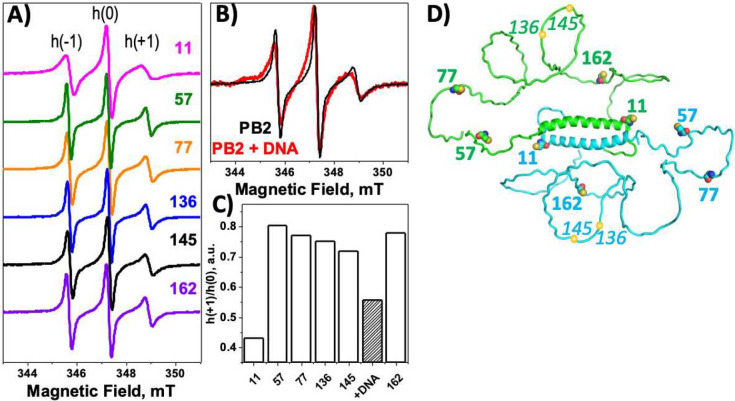
Continuous wave EPR spectroscopy of PALB2-DBD. **(A)** cwEPR spectra of PALB2-DBD labeled at different positions indicated by the number on the right. **(B)** Overlay of PALB2-DBD spectra labeled at position 145 in free (black) and DNA-bound (red) states. **(C)** h(+1)/h(0) values for each position of the attached label. Texture-filled bar corresponds to the signal from position 145 in the presence of DNA. No changes of the ratio were observed on DNA binding for other positions. **(D)** Hypothetical conformation of PALB2-DBD dimer generated by the AlphaFold program. Monomers are shown in cyan and green. Cysteine positions used for labeling are marked with spheres and aa numbers.

These results do not completely rule out the potential formation of additional α-helixes on coiled-coil–mediated oligomerization of PALB2-DBD. As IDRs are characterized by high flexibility, we analyzed the local flexibility of different regions using continuous wave electron paramagnetic resonance (cwEPR) spectroscopy, which is particularly informative for structural characterization of IDPs and their interactions (Fig. 2)^65,66^. Four cysteines in human PALB2-DBD were alternatively mutated to create single cysteine variants at positions 11, 57, 77, and 162 (Fig. 2D). Two additional cysteines were introduced in the vicinity of the major DNA-binding site (S136C, S145C). The nitroxide-containing spin label 1-oxyl-2,2,5,5-tetramethyl-d3-pyrroline-3-methyl methane thiosulfonate (MTSSL) was covalently attached to the cysteine thiol group for EPR analysis. The EPR spectrum was measured for each mutant alone and for positions 11, 136, and 145 in the presence of ssDNA (Fig. 2B). Most spectra correspond to a highly flexible conformation of local peptide in the label vicinity. Values of the h(+1)/h(0) ratio correspond to those for other IDPs (Fig. 2C)^66^, with the exception of position 11 at the beginning of the PALB2-cc (a lower value corresponds to a more rigid structure near the coiled-coil region). The addition of dT_50_ causes a notable change in flexibility at position 145 immediately next to the major DNA-binding site (146–149 aa). This region is still more flexible even in the presence of DNA than at position 11. There was no change in flexibility of the region around positions 11 and 136. Thus, DNA binding does not affect complex mobility or structural flexibility beyond the immediate vicinity of the DNA-binding site. High flexibility of the probed regions further confirms a lack of any secondary structures formed inside the Δ40-DBD sequence, even within the oligomeric PALB2-DBD and in the DNA-bound state. The combined results indicate that PALB2-DBD has an intrinsically disordered and highly flexible structure beyond the N-terminal α-helix.

### PALB2-DBD dimer represents a compact molten globule

IDPs have different functionally relevant structural organizations ranging from compact molten globule (MG) structures to extended preMG and random coil (RC) conformations. MG structures have a relatively small hydrodynamic volume (*V*_*h*_) that are only 2–3 times larger than the volume of a folded globular structure of the same molecular weight^67^. RC structures have up to 10 times larger *V*_*h*_ than that of folded protein.

Since the PALB2-DBD structural organization is further complicated by oligomerization, we initially evaluate the compaction of monomeric Δ40-DBD using size exclusion chromatography (SEC) to compare the elution time of the protein in a DNA-binding buffer with that of the protein in the presence of the strong denaturant guanidinium hydrochloride (GdmHCl), which forces RC formation (Extended Data Fig. 2A). The protein elutes significantly earlier in 6 M GdmHCl than in non-denaturing DNA-binding buffer, suggesting that Δ40-DBD has a compact MG conformation. The elution volume under nondenaturing conditions corresponds to a globular protein with a molecular weight only twice that of Δ40-DBD (Extended Data Fig. 2B). These combined results suggest that the Δ40-DBD region lacks secondary structure elements but forms a relatively compact MG structure.

We previously reported an apparent tetrameric form of PALB2-DBD based on SEC and DNA-binding stoichiometry^32^. Considering a disordered structure of PALB2-DBD that leads to SEC overestimation of the molecular weight, we analyzed PALB2-DBD using two alternative methods: analytical centrifugation with a sedimentation velocity method and multiangle light scattering (MALS). PALB2-DBD alone sediments as a dimer even at relatively high protein concentration (Extended Data Fig. 3A). The molecular weight was directly measured using MALS combined with SEC at two different solution conditions with high (0.5 M) and low (0.16 M) NaCl concentrations (Extended Data Fig. 3B, C). Two salt concentrations were used because PALB2-DBD is more soluble and less prone to aggregation in high salt buffer (0.5–1.0 M NaCl), whereas DNA binding is strongly inhibited by high salt concentrations^32^. MALS results unequivocally confirmed that PALB2-DBD is a dimer under both conditions.

**Fig. 3 |:**
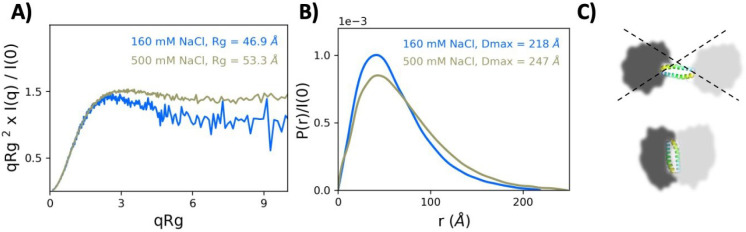
SAXS/MALS analysis of PALB2-DBD. **(A)** SAXS data for high (green) and low (blue) concentration salt buffers, visualized as a dimensionless Kratky plot. Data were binned logarithmically by factor of 4 for visual clarity. **(B)** Pairwise distance distribution functions [*P*(*r*)] from SAXS measurements of PALB2-DBD in low (blue) and high (green) concentration salt buffers. **(C)** Two hypothetical models of IDRs attached to opposite ends of coiled-coil linker. A single domain length in *P*(*r*) distribution (B) rules out a dumbbell model (top) and suggests a compact dimer conformation (bottom).

Structural organization of the PALB2-DBD dimer was characterized using small angle X-ray scattering (SAXS) analysis which reveals important structural features of proteins in solution and is particularly valuable for IDPs that are not amenable to other structural methods. We analyzed PALB2-DBD in two different buffer conditions with 0.16 M or 0.5 M NaCl NaCl using SEC-MALS-SAXS (Extended Data Table 1, Extended Data Figs. 4, 5). These analyses resulted in typical IDP scattering patterns (Fig. 3A).

Two IDRs in the PALB2-DBD dimer are located at opposite ends of 40 Å-long antiparallel coiled-coil structure and can form two independent or spatially distinct MGs. However, only one dominant length scale is observed in the pairwise distance distribution [*P*(*r*)] measured in SAXS (Fig. 3B), ruling out the dumbbell model with two distinct MGs (Fig. 3C). The small radius of gyration (*R*_*g*_) values obtained in SAXS experiments also indicates a compact organization of the entire dimer (Fig. 3A). These combined data suggest that each monomer forms a compact MG stabilized by intramolecular interactions. Similar interactions between two IDRs are likely to contribute to synergistic dimerization with a coiled-coil interaction, thereby leading to a compact structure of the entire dimer.

**Fig. 4 |:**
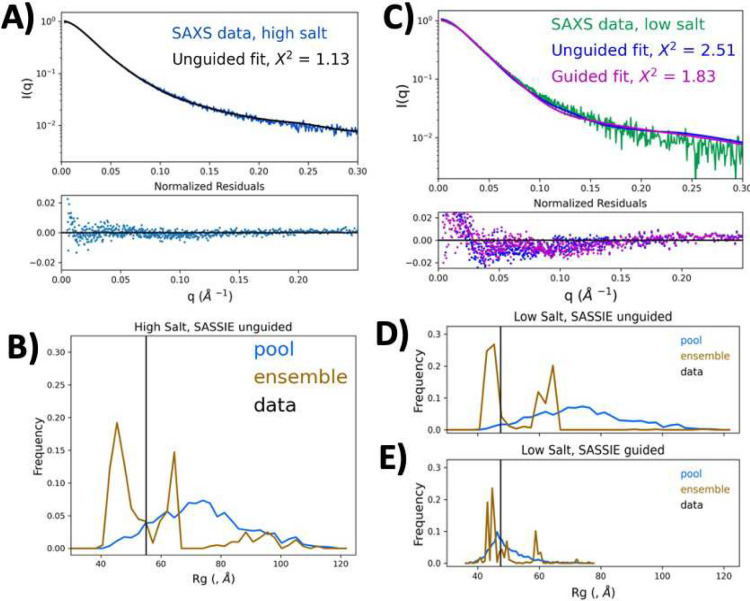
Modeling of the PALB2-DBD structural ensemble. **(A)** Fit of SAXS-derived experimental distance distribution in 0.5 M NaCl buffer with theoretical distribution calculated from unguided SASSIE models. Normalized residual errors are shown at the bottom. **(B)** R_g_ distribution of the entire pool of 17,000 structures (blue), experimental R_g_ (black bar), and distribution of R_g_ of the best-fit pool structures (brown). **(C)** Similar to (A) but for SAXS data obtained from samples in 0.16 M NaCl buffer fit to unguided models (blue) and guided models with restricted R_g_ (magenta). **(D)** R_g_ distributions for unguided model pools. **(E)** R_g_ distributions for guided model pools.

To obtain further insight into PALB2-DBD structural organization using experimental SAXS results, we performed molecular structure modeling using the SASSIE program^68^. Random structures were generated using the Complex Monte-Carlo algorithm in SASSIE with fixed N-terminal α-helixes and a dimeric coiled-coil interface (aa 11–40) as determined by NMR^45^. The starting model of a dimer was generated by the AlphaFold program using the ColabFold Google server^69^ (Fig. 2D). The resulting trajectory yielded 17,353 structures, from which a subensemble of structures (selected using GAJOE^70^) provided a good fit (χ^2^=1.14) to the experimental data of the system in 0.5 M NaCl buffer (Fig. 4A). The poll of the best-fit ensemble is bimodal with the majority with lower *R*_*g*_=45 Å and a second population with *R*_*g*_=62 Å. The experimental Guinier *R*_*g*_=54.9 Å is significantly lower than the average *R*_*g*_=75 Å of the entire pool of all randomly generated structures. We analyzed data using a molecular form factor (MFF) fitting protocol developed specifically for IDPs (http://sosnick.uchicago.edu/SAXSonIDPs)^71,72^. The result yields a Flory exponent ν=0.52, which describes a polymer dimension dependence on its length (*R*_*g*_~N^ν^). This value of ν is lower than those for typical IDPs in good solvent (~0.54–0.55)^71,72^, and suggests a relatively compact overall conformation, assuming that this system behaves relatively similarly as a single-chain IDP with a total length of 400 aa due to the almost end-to-end dimerization.

Fitting data obtained at a low salt concentration (0.16 M NaCl) was more challenging. Initial unguided SASSIE modeling resulted in relatively poor fit with χ^2^=2.5 (Fig. 4C), seemingly due to the propensity of the trajectory to more extended structures. A guided fit with an imposed restriction on average *R*_*g*_ during modeling resulted in χ^2^=1.83, which was still worse than that for a high salt buffer. A similar bimodal distribution of theoretical *R*_*g*_ was obtained even with guided modeling (Fig. 4E). Therefore, we used the AllosMod-FoXS program^73,74^ with concurrent energy landscape modeling (i.e., multiple different starting points) to perturb the structure to fit the experimental data (Extended Data Fig. 6). We used several different starting models to generate 100 concurrent trajectories, each generating 100 models. Pooling all structures from the two trajectories and GAJOE fitting of a subensemble^75,76^ resulted in a better fit for both experimental datasets. Although this method does not allow fixing of the dimeric interface, the resulting structures retained an overall similar orientation of the α-helices. This modeling also resulted in bimodal *R*_*g*_ distribution of best-fit structural ensembles.

These data further support a highly compact dimeric PALB2-DBD structure of two IDRs. Notably, compaction parameters at 0.5 M NaCl (*R*_*g*_=58.4 Å and Flory scaling exponent ν=0.52, Extended Data Table 2) are more similar to the corresponding values of those reported for an unstructured PNt domain with slightly smaller single chain size (335 aa) than that of the PALB2-DBD dimer^71^. The *R*_*g*_ (47.5 Å) and ν (0.47) values are lower in the low salt condition than in the high salt condition, suggesting a role for polar interactions in structure compaction. The *R*_*g*_ values calculated from fitting the MFF (55.0 Å and 49.1 Å for the high and low salt conditions, respectively) are slightly larger than the reference Guinier *R*_*g*_ values but are relatively close in both cases, and thus would not affect our interpretation of the results.

### Single-molecule FRET analysis reveals that PALB2-DBD dynamically condenses DNA

The recombination mechanism depends on conformational changes of DNA bound to recombinase. RecA-like recombinases form nucleoprotein filaments stretching out ssDNA^77–79^. Rad52 wraps ssDNA around a toroidal oligomeric structure^24,25,80^. Our previous intensity-based Förster resonance energy transfer (FRET) experiment suggested a Rad52-like mechanism where protein interaction led to increased FRET between Cy3 and Cy5 dyes located at the ends of doubly labeled dT_40_ or dT_70_ oligonucleotides^32^. Titration by PALB2-DBD led to higher FRET efficiency, thus resembling the results of a similar experiment with Rad52^80^. However, these measurements were performed on an ensemble averaged in a fluorimeter, so the molecular species and sequence of events that generated the result could not be precisely identified, particularly considering the disordered nature of PALB2-DBD and oligomerization.

**Fig. 5 |:**
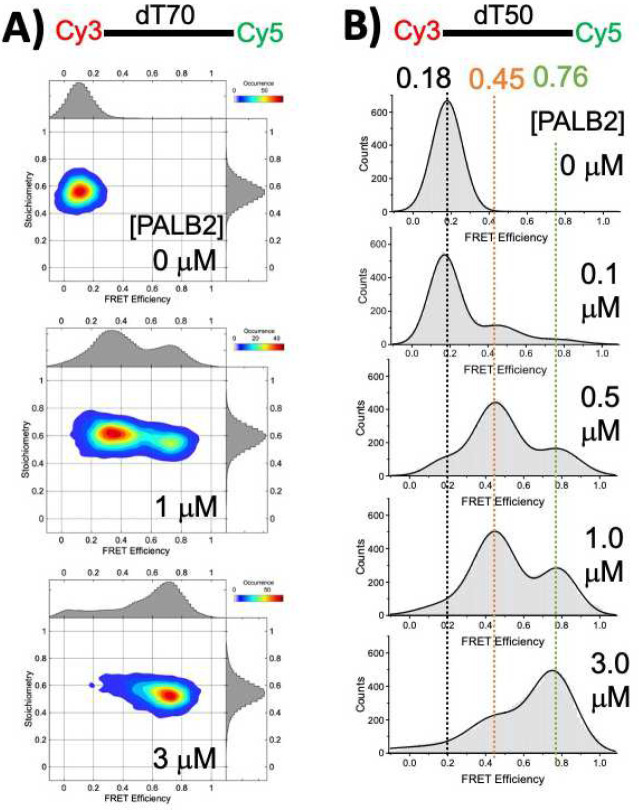
Bimodal DNA compaction revealed by confocal smFRET. **(A)** 2D plots of stoichiometry versus FRET efficiency for 100 pM Cy3-dT_70_-Cy5 alone and in the presence of 1.0 μM and 3.0 μM PALB2-DBD. **(B)** FRET histograms (grey) of 100 pM Cy3-dT_50_-Cy5 titrated by PALB2-DBD. Black lines show fitting of data using three species as Gaussian distributions. Protein concentrations are shown on each graph. Mean FRET values of major peaks are shown with color corresponding to each peak.

To address the limitation of our previous studies, we performed comparable experiments utilizing single-molecule FRET (smFRET). We used a confocal setup to determine the FRET efficiency of freely diffusing single DNA molecules labeled with the FRET pair Cy3/Cy5 in the absence and presence of PALB2-DBD. We used Pulsed interleaved excitation (PIE) to calculate the stoichiometry for each molecule, as the PIE value enables the identification of molecules containing the correct 1:1 ratio of donor and acceptor (Fig. 5A) and the detection of other photophysical phenomena such as bleaching, blinking, and protein-induced fluorescent enhancement (PIFE)^81–84^. Measurements were performed with several ssDNA substrates with fluorescent dyes placed at different distances: dT_50_ with Cy3 at the 5′ end and Cy5 at positions 25 or 50, and dT_70_ labeled at the 5′ and 3′ ends. Cy3-dT_70_-Cy5 and Cy3-dT_50_-Cy5 substrates in solution show a single, symmetric distribution with mean FRET of 0.11 (Fig. 5A) and 0.18 (Fig. 5B), respectively, in agreement with published results^85^. The addition of PALB2-DBD significantly increased mean FRET, thereby confirming observations from our previous studies^32^. Two distinct FRET populations were observed in the presence of PALB2, corresponding to two different states of DNA bound to the protein, one significantly more condensed than the other with mean FRET of 0.34 and 0.71 in case of Cy3-dT_70_-Cy5 (Fig. 5A). The mean FRET value for each peak is invariant for a given DNA fragment, whereas the population of each state changes upon titration by PALB2-DBD. A medium FRET state of Cy3-dT_70_-Cy5 is a predominant species at 1 μM PALB2-DBD, whereas high FRET is a major peak at 3 μM PALB2-DBD (Fig.5A). Titration of dT_50_ by PALB2-DBD (Fig. 5B) revealed similar distributions of two populations of invariant mean FRET peaks of 0.45 and 0.79, with a high FRET peak becoming more predominant at high protein concentration. These results suggest that PALB2-DBD binds to multiple sites within the same DNA strand, thereby dynamically controlling its conformation. They also suggest that the PALB2-DBD concentration is an important factor in controlling DNA conformation.

**Fig. 6 |:**
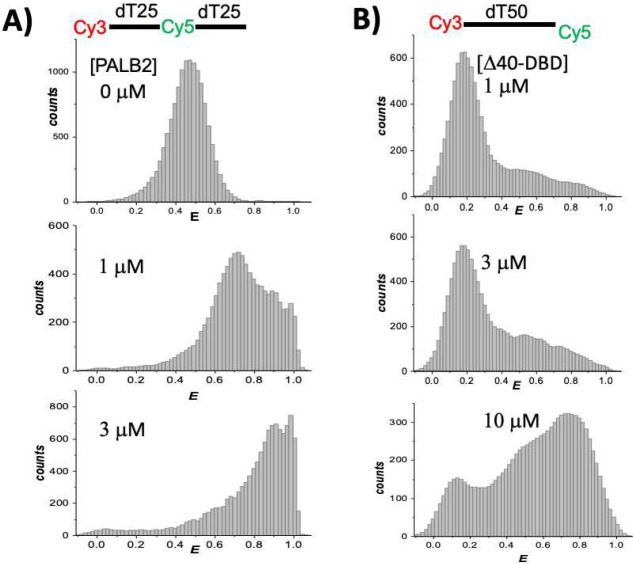
Single-molecule FRET measurements of ssDNA compaction. **(A)** FRET histograms of 100 pM dT_50_ with Cy3 placed at the 5′ end and Cy5 attached at position 25 of the dT50 substrate alone and in the presence of 1 μM and 3 μM PALB2-DBD. **(B)** FRET histograms of Cy3-dT_50_-Cy5 titrated by Δ40-DBD. Protein concentrations are shown on each graph.

A similar trend was observed for a dT_50_ substrate with Cy3 and Cy5 labels placed 25 nucleotides apart (Fig. 6A). Mean FRET for free DNA is 0.45, the major peak at 1 μM PALB2-DBD is at FRET of 0.71, and the peak for 3 μM PALB2-DBD is at FRET of 0.89. DNA compaction is significant for this substrate as well, although it is challenging to deconvolute two separate peaks at each protein concentration due to the initially high FRET for free DNA. A similar trend of DNA compaction proportional to the number of nucleotides between labels is observed for all three cases. For example, changes in FRET between the peak of free DNA and the first peak of DNA in complex with PALB2-DBD for distances of 70, 50, and 25 nucleotides were from 0.11 to 0.34, from 0.18 to 0.45, and from 0.5 to 0.7, respectively. These results ruled out a Rad52-like wrapping model of DNA binding, in which the FRET change between remote ends should be greater than that between labels situated closer in the nucleotide sequence.

The broad distributions of each peak suggest a random coil model of ssDNA conformation, in which the average distance between ends is restricted due to interactions with two major DNA-binding sites in a compact PALB2-DBD dimer. In this model, two random parts of ssDNA should interact with two DNA-binding sites situated within an average *R*_*g*_ distance from each other. This model explains an invariant position of the peak with an intermediate mean FRET. The mechanism of an additional DNA compaction corresponding to a high FRET peak remains to be further investigated. One hypothesis is that an additional compaction is caused by a higher oligomeric state of PALB2-DBD. To evaluate the role of PALB2-DBD oligomerization in DNA compaction, we tested a Δ40-DBD variant lacking the coiled-coil dimerization interface using Cy3-dT_50_-Cy5 (Fig. 6B). DNA compaction also was observed in this case, albeit at higher protein concentrations, reflecting a significantly reduced DNA binding affinity^32^. There was a fraction of free DNA in solution even at 10 μM protein. FRET distributions were significantly broader and more heterogeneous corresponding to multiple species of DNA conformations. The lack of two distinct peaks in the case of Δ40-DBD indirectly supports oligomerization-dependent DNA compaction. In this case, the Δ40-DBD will form oligomers by addition of a single monomer (dimers, trimers, tetramers) resulting in a broad distribution of DNA conformations, while PALB2-DBD forms dimers and, potentially, tetramers resulting in a more distinct FRET values.

The tetramerization hypothesis is supported by a previously observed 1:4 stoichiometry of DNA binding in fluorescence polarization and ensemble FRET assays^32^, but is contradicted by a dimeric state of PALB2-DBD measured by analytical ultracentrifugation (AUC) and MALS. We repeated sedimentation velocity experiments with PALB2-DBD in the presence of various ssDNA substrates (Extended Data Fig. 7A, B). DNA was labeled with Cy3 to distinguish its position from that of protein-only. Measurements were repeated at two different protein concentrations corresponding to DNA:protein molar ratios of 1:2 and 1:4. In the case of Cy3-dT_50_, two DNA peaks were observed. A larger peak overlapped with that of the PALB2-DBD dimer and a smaller peak with a molecular weight comparable to that of the tetrameric PALB2-DBD. Although free Cy3-dT_50_ has similar sedimentation as that of the PALB2-DBD dimer, most DNA should be in the protein-bound form because the concentration of components is several times above the DNA binding K_d_. To distinguish free from bound DNA, we repeated the experiment with shorter Cy3-dT_25_ (Extended Data Fig. 7B). There was no peak corresponding to free DNA in solution at either the 1:2 or 1:4 stoichiometry. We also observed a small peak corresponding to a tetrameric structure. These data suggest that DNA readily interacts with PALB2-DBD dimers and stimulates partial tetramerization of PALB2-DBD. The combined results also rule out the independent binding of two dimers to neighboring parts of a long DNA molecule because similar tetrameric structures were formed with 25- and 50-mer ssDNA. These results only partially support the hypothesis that the high FRET state corresponds to DNA bound to tetramers, since there is only a minor population of tetramers in AUC, whereas it is a major peak in smFRET experiments at similar protein concentrations (3 μM in smFRET and 4 and 8 μM in AUC). The DNA:protein stoichiometries differ in the smFRET and AUC experiments, with a several orders of magnitude larger protein excess versus DNA in smFRET but only 2–4 fold excess in AUC. We repeated smFRET measurements with the addition of 0.5 μM unlabeled DNA (Extended Data Fig. 7D). Higher concentration of total ssDNA significantly reduced the high FRET efficiency fraction, approximately recapitulating the ratio between dimeric and tetrameric species in AUC experiments. Therefore, PALB2-DBD tetramerization requires a large excess of free protein relative to DNA in solution.

## Discussion

Our data indicate that PALB2-DBD is an intrinsically disordered domain that forms a dimer stabilized by a coiled-coil interface between N-terminal α-helices and interactions between IDRs. Although PALB2-DBD is comprised of a low complexity sequence, it was unexpected that an unstructured protein can support an elaborate multisubstrate and multistep mechanism of strand exchange. The Δ40-DBD domain has a balanced composition of hydrophobic, positively, and negatively charged amino acids (Extended Data Fig. 1D, E) that can undergo context-dependent folding affected by protein-protein or DNA-protein interactions^86,87^. Analyses using the CIDER server^50^ classified Δ40-DBD at the boundary between “Janus sequences: Collapsed or expanded–context dependent” and “Weak polyampholytes & polyelectrolytes: Globule & tadpoles”. Analysis of this protein by the flDPnn platform (http://biomine.cs.vcu.edu/servers/flDPnn/), designed for the accurate intrinsic disorder prediction with putative propensities of disorder functions^88^, further supports the excessive disorder-based interactivity of PALB2-DBD predicting multiple regions with a strong propensity for protein binding as well as DNA and RNA interactions (Extended Data Fig. 1 H). Therefore, it seems that the intrinsically disordered nature of PALB2-DBD defines not only its binding promiscuity, making this protein capable of interaction with multiple proteins and nucleic acids, but a novel for IDP functionality of DNA and RNA strand exchange.

SEC and SAXS results revealed that each monomer (Δ40-DBD) and the entire PALB2-DBD dimer has a compact structure not typical for scaffold structures interacting with multiple proteins. This compaction is likely achieved through favored intermolecular and intramolecular interactions between two domains and does not represent a collapsed state of disordered protein chains. The Flory scaling exponent describing a polymer dimension dependence on its length (ν) is around 0.5, which is expected for a non-self-avoiding random coil and corresponds to a Θ solvent where intermolecular and solvent interactions counterbalance each other^72^. A noticeable reduction of *R*_*g*_ and ν in a low salt buffer suggests that polar intermolecular interactions strongly contribute to PALB2-DBD compaction. The same intramolecular interactions are likely to stabilize and compact the entire dimer. The interaction between two IDRs is weak, since Δ40-DBD is monomeric in solution. However, they are synergistic with the coiled-coil interactions. The dimerization of isolated coiled-coil helices is rather weak, with *K*_*d*_=80 μM as reported by an NMR titration experiment^45^, whereas the dimer remains intact in our experiments even during nonequilibrium gel filtration at 100 times lower concentrations. A single dominant length scale observed in the pairwise distance distribution [*P*(*r*)] measured in SAXS (Fig. 3B) further supports interactions of entire domains and not only coiled-coil helixes. It is tempting to hypothesize that protein-protein interactions by IDRs can contribute to heterooligomerization within DNA repair complexes. For example, PALB2 binds BRCA1 through the same N-terminal α-helix that forms a coiled-coil interface with the BRCA1 α-helix^49,89^, and these interactions can be strengthened by attractions between IDRs of two proteins.

The conformation of DNA bound to protein defines the mechanism of strand exchange. RecA-like recombinases stretch ssDNA, keeping nucleotide triplets with exposed bases in a B-form-like geometry while bending the DNA helix and transiently separating the two strands^90^. The mechanism of Rad52 recombination is less well understood. It wraps ssDNA around the toroidal oligomer backbone, keeping bases exposed for base pairing^80,91^. Binding of dsDNA along the toroidal oligomer can potentially distort the helical structure, leading to transient strand separation and strand exchange. We observed significant compaction of ssDNA on PALB2-DBD binding, ruling out a RecA-like mechanism. Changes of FRET efficiency on PALB2-DBD binding are proportional to the number of nucleotides between labels, thus ruling out a wrapping model. These combined results indicate that the mechanism of PALB2-DBD interaction with ssDNA differs from those of RecA and Rad52.

**Fig. 7 |:**
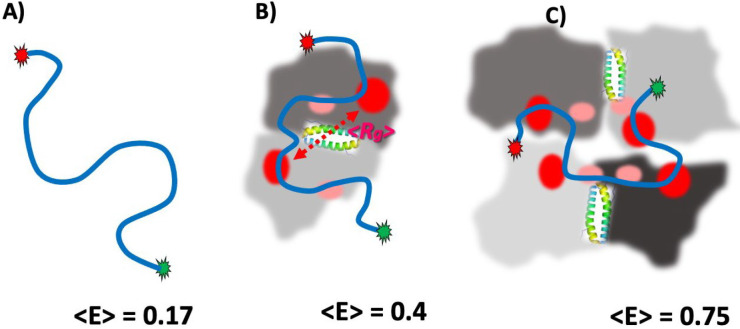
Model of ssDNA compaction by PALB2-DBD dimer and tetramer. **(A)** Cy3-ssDNA-Cy5 (blue line with red and green marks for fluorophore positions) in solution. **(B)** Cy3-ssDNA-Cy5 bound to PALB2-DBD dimer. Two monomers are shown by dark and light grey. Major DNA-binding sites, red; minor DNA-binding sites, pink. **(C)** Cy3-ssDNA-Cy5 bound to PALB2-DBD tetramer. Each dimer forms around coiled-coil interface; the interaction between the two dimers is mediated by bound DNA.

Wide FRET distributions of PALB2-DBD–bound ssDNA and the invariant positions of observed peaks suggest random coil folding with additional distance restrictions. The interaction of ssDNA at two random positions with two DNA-binding sites of the dimer (and potentially with minor DNA-binding sites) situated within an average *R*_*g*_ distance from each other should lead to the observed distribution (Fig. 7). The results indicate approximately two-fold DNA compaction, with a medium FRET value of Cy3-dT_50_-Cy5 bound to PALB2-DBD equal to that of unbound DNA with 25 nucleotides between Cy3 and Cy5.

The mechanism behind the formation of high FRET peaks for each substrate is less clear. We hypothesize that this conformation can be formed by interaction with tetrameric PALB2-DBD. Similar attraction forces between IDRs, which stabilize the dimer, can synergistically stimulate interactions between two dimers along with DNA binding. Additional compaction of DNA bound to a larger complex can be achieved by condensation of DNA-binding sites around a ssDNA molecule. The physiological role of PALB2-DBD tetramerization remains unclear considering the required stoichiometric excess of protein suggested by our results and the large size of the full-length protein and of its functional complexes with even larger BRCA2 and BRCA1. However, attractions between IDRs can stimulate formation of heterooligomeric complexes with disordered parts of BRCA1/BRCA2 and other protein partners or the formation of DNA-repair condensates through liquid-liquid phase separation (LLPS). The probability of forming a droplet state through LLPS (p_LLPS_) for PALB2-DBD and PALB2 is very high, as calculated using FuzDrop analysis (0.9622 and 0.9412, respectively, Extended Data Fig. 8). Since these values significantly exceed the 0.6 threshold, both PALB2-DBD and PALB2 are considered as potential droplet-drivers, which can spontaneously undergo liquid-liquid phase separation^55^. Therefore, the observed limited oligomerization of PALB2-DBD may reflect its ability to stimulate formation of biocondensates under certain conditions, which remained to be identified.

Our DNA compaction results suggest the following mechanistic hypothesis: the PALB2 dimer destabilize a DNA helix by imposing a similar compaction force on dsDNA as observed for ssDNA, and two DNA chains can reanneal either with each other or with a homologous ssDNA bound to the same protein complex. We previously demonstrated that PALB2-DBD does not unwind DNA in ensemble solution experiments; by contrast, it stimulates complementary strand annealing^32^. Therefore, distortion of the double helix should be transient while bound to the protein, and concurrent with binding an additional ssDNA. The disordered structural organization of the protein may be advantageous for transient interactions. Iterative cycles of distortion-mediated strand separation and annealing can be described as a chaperone-like mechanism. This mechanism could explain a slow equilibrium process undergoing multiple association/dissociation cycles and lack of directionality, in contrast to RecA-like recombinases or Rad52^17,32^.

Chaperon activities are critical for RNA metabolism due to the complexity, diversity, and the dynamic nature of RNA structures and their complexes. Various mechanisms are supported by a wide spectrum of proteins ranging from ATP-dependent helicases to small IDPs^92,93^. *Escherichia coli* StpA protein promotes RNA strand annealing and strand displacement^94^, similar to those reported for PALB2-DBD^32^. SptA is a small globular protein that forms dimers required for these activities. Other examples include the structured globular FANCA protein of the Fanconi anemia (FA) complex^28^ and limited reports of D-loop formation by FET proteins critical for RNA metabolism, specifically pro-oncoprotein TLS/FUS and the human splicing factor PSF/hPOMp100^29–31^. The latter proteins include structured domains (RNA recognition motif RRM and zinc finger), sequences of low complexity, and prion-like RGG domains implicated in higher-order self-assemblies. There are examples of disordered RNA chaperones composed of positively charged peptides that promote the formation of compact nucleic acid conformations by acting as polypeptide counterions to overcome repulsive electrostatic interactions of RNA or DNA chains^62–64^. Chaperone activities are not implicated in DNA metabolism due to significantly more restricted conformational space and size of chromosomal fragments. However, multiple DNA metabolism processes (e.g., repair of replication forks collided with transcription complexes and replication fork reversal) require the formation of transient multichain DNA and RNA-DNA structures and other higher-order nucleic acid interactions^95–97^ that may benefit from strand exchange activity of PALB2-DBD or similar unstructured domains of DNA-repair proteins. The fact that PALB2 resides at the promoter regions of highly transcribed genes under nondamaging conditions^98^ further supports this functional hypothesis. Moreover, DNA-binding proteins are enriched in disordered domains/regions, have higher disordered content in eukaryotes^99^, and many IDRs coincide with DNA-binding domains^100^. Such regions may simply increase the chromatin affinity by transient DNA binding without forming a strong roadblock on DNA, except in cases of structure-specific interactions like nucleolin-G–quadruplex recognition^101^. The described here novel DNA-chaperone mechanism of PALB2-DBD suggests that DNA-binding IDRs may have evolved to perform more complex reactions using an evolutionarily inexpensive functional unit that facilitates formation and resolution of transient multichain DNA/RNA intermediates during chromatin repair. Although this proposed reaction is more complex than others reported for IDRs, it depends on simple structural requirements and similar IDRs can be incorporated into larger proteins (e.g., DNA-binding regions in scaffold BRCA1^100,102,103^ and BRCA2^104,105^). Extended Data Fig. 9 provides some support to the validity of this hypothesis by representing several disorder-centric features of the DNA-binding domain of BRCA1 which show rather impressive overall similarity to the similar profiles generated for PALB2-DBD (*cf.* Extended Data Fig. 1). These disordered domains can function synergistically with RAD51^32,40,106^, or they can independently perform a required reaction without recruiting additional specialized factors (e.g., RAD52) and without forming stable presynaptic RAD51 filaments. Furthermore, such IDRs often have high propensity for LLPS formation, suggesting that they can stimulate formation of DNA repair condensates and function within such condensates. For example, sequence analysis of BRCA1 DBD region in vicinity of PALB2-binding site (Extended Data Fig. 9) revealed a high probability of droplet formation by this region. This is a very compelling possibility, as the LLPS-driven formation of various membrane-less organelles (MLOs) and biomolecular condensates is considered now as one of the crucial organizing principles of the intracellular space responsible for regulation and control of numerous cellular processes^107^. Among these MLOs related to the subject of this study are the DNA repair foci, formation of which represents a part of the DNA damage response ^108^,^109^,^110^,^111,112^; Promyelocytic leukemia Nuclear Bodies (PML NBs) that are physically associated with chromatin^113^ and related to the chromatin function; liquid condensates on DNA/RNA matrices containing related proteins and G-quadruplexes (G4s) and responsible for reparation, transcription, genome integrity maintenance, and chromatin remodeling ^114^; nuclear condensates containing polycomb group (PcG) proteins that contribute to the reshaping of chromatin architecture under the physiological and pathological conditions^115^; and intrinsic chromatin condensates related to various chromatin-centric processes, such as transcription, loop extrusion, and remodeling^116^ to name a few. Therefore, it is likely that the LLPS-driven formation of biomolecular condensates containing this protein and nucleic acids can play a role in complex functionality of PALB2-DBD and similar regions of other DNA-binding IDRs,

## Materials and Methods

### Cloning and purification

PALB2 N-terminal fragments (1–195 I195W and 1–295 K295W) were cloned into a pSMT-MBP plasmid containing an N-terminal 6×His-SUMO and a C-terminal MBP tag using Gibson assembly. The pSMT-MBP plasmid was created from a pET28b^+^–based pSMT3 plasmid (gift of Dr. R. A. Kovall, University of Cincinnati) by insertion of a TEV cleavage site and MBP at the ORF C-terminus. Mutations were introduced using the Stratagene QuikChange protocol. All constructs were fully sequenced and verified. Constructs were transformed into BL21* cells for expression. Cell cultures were grown in TB to OD_600_=1.6, and protein expression was induced by 0.2 mM IPTG overnight at 16°C. Cell suspensions were centrifuged at 4,000 rpm for 15 min. Cell pellets were resuspended in lysis buffer (25 mM HEPES pH 8.0, 1 M NaCl, 10% glycerol, 2 mM CHAPS, 1 mM TCEP, 0.3% Brij35, 2 mM EDTA, 1 mM PMSF), frozen in liquid nitrogen, and stored at −80°C. Frozen cell suspension was thawed and lysed with lysozyme at 0.25 mg/ml for 20 min at room temperature, followed by three rounds of sonication (50% output and 50% pulsar setting for 4 min). Cell debris was removed by centrifugation at 30,600 *g* for 45 min. Supernatant was loaded onto an amylose resin column (New England Biolabs) equilibrated with binding buffer (25 mM HEPES pH 8.0, 1 M NaCl, 10% glycerol, 2 mM CHAPS, 1 mM TCEP). Resin was washed with binding buffer containing 0.05% NP40 and 1 mM EDTA, followed by a second wash with low-salt buffer (50 mM NaCl, 25 mM HEPES pH 8.0, 1 mM TCEP, 2 mM CHAPS, 0.2% NP40, 1 mM EDTA). Protein was eluted in a binding buffer containing 20 mM maltose. SUMO and MBP tags were cleaved using Ulp1 and TEV proteases, respectively. Protein was diluted five-fold with binding buffer without NaCl to bring the NaCl concentration to 200 mM, loaded onto a Hi-Trap heparin affinity column (2×5 ml, GE Life Sciences), and eluted with a salt gradient (200–800 mM NaCl). Protein eluted from the column in the ~600 mM NaCl fraction. Gel filtration was performed using a Superdex-200 10/300GL column (GE Life Sciences).

### Circular dichroism (CD)

A Zeba spin desalting column was used to exchange protein buffer to buffer containing 10 mM NaPO_4_, 150 mM NaF, and 2 mM CHAPS. Samples were centrifuged at 16,000 *g* to remove aggregates, and the final concentration was verified. Measurements were performed using a Jasco J-715. Four samples were used for CD measurements: (1) 30 μM PALB2-DBD, (2) 30 μM Δ40-DBD, (3) 30 μM PALB2-DBD with 30 μM dT_50_, and (4) 30 μM Δ40-DBD with 30 μM dT_50_. Each spectrum is an average of 10 scans.

### Electron paramagnetic resonance (EPR) analysis

Monocysteine PALB2 mutant proteins were labelled with MTSSL [S-(1-oxyl-2,2,5,5-tetramethyl-2,5-dihydro-1H-pyrrol-3-yl)methyl methanesulfonothioate] in a sample free of reducing agents. Unbound label was separated on a Superdex-200 10/300 gel filtration column. Spectra were measured using 20 μM protein in 20 mM Tris-acetate pH 7, 100 mM NaCl, 10% DMSO, and 5% glycerol with or without 80 μM dT_40_. Continuous wave EPR spectroscopy (cwEPR) was performed on an EMX spectrometer operating at X-band frequency (9.77 GHz) at room temperature. A dielectric resonator (ER4123D) and 25 μl borosilicate glass capillaries were used. The center field was set to 3474 G and the sweep width was 150 G. The modulation amplitude, microwave power, and resolution were set to 1.6 G, 2 mW, and 1125 points, respectively.

### SEC-MALS-SAXS analysis

SAXS was performed at BioCAT (beamline 18ID at the Advanced Photon Source, Chicago) with in-line size exclusion chromatography (SEC) to separate sample from aggregates and other contaminants, thereby ensuring optimal sample quality. Multiangle light scattering (MALS), dynamic light scattering (DLS), and refractive index measurement (RI) were used for additional biophysical characterization (SEC-MALS-SAXS). The samples were loaded on a Superdex 200 Increase 10/300 GL column (Cytiva) run by 1260 Infinity II HPLC (Agilent Technologies) at 0.6 ml/min. The flow passed through (in order) the Agilent UV detector, a MALS detector and a DLS detector (DAWN Helios II, Wyatt Technologies), and an RI detector (Optilab T-rEX, Wyatt). The flow then passed through the SAXS flow cell. The flow cell consists of a 1.0 mm ID quartz capillary with ~20 μm walls. A coflowing buffer sheath is used to separate samples from the capillary walls, helping prevent radiation damage (Kirby et al., 2016). X-ray data were collected using a 150 (h) × 25 (v) (μm) 12 keV beam, and scattering intensity was recorded using an Eiger2 XE 9M (Dectris) photon-counting detector. The detector was placed 3.6 m from the sample giving access to a *q-*range of 0.0029 Å^−1^ to 0.417 Å^−1^, with the momentum transfer vector *q* defined as 𝑞 = (4*π/λ*)sin(*θ*). Exposures (0.5 s) were acquired every 1 s during elution, and data were reduced using BioXTAS RAW 2.1.1 (Hopkins et al., 2017). Buffer blanks were created by averaging regions flanking the elution peak and subtracted from exposures selected from the elution peak to create the I(*q*) vs *q* curves used for subsequent analyses. Molecular weights and hydrodynamic radii were calculated from the MALS and DLS data, respectively, using ASTRA 7 software (Wyatt).

### Confocal smFRET analysis

All samples were prepared in buffer (0.16 M NaCl, 20 mM HEPES pH 8.9, 1 mM TCEP, 0.003% Tween 20) and centrifuged at 40,000 rpm for 20 min before the experiment. FRET measurements of freely diffusing single molecules were performed with a confocal microscope [MicroTime 200 (PicoQuant)] as described previously^83,117^. The donor and acceptor were excited by light from 532 nm and 638 nm lasers, respectively. A pulsed interleaved excitation (PIE) setup was used with a pulse rate of 40 MHz to alternate donor and acceptor excitation. PIE reports the status of both donor and acceptor fluorophores by sorting molecules based on relative donor:acceptor stoichiometry (S) and apparent FRET efficiency (E) as described previously^81,84,118^. Measurements were performed 25-μm deep in the solution using a laser power of ~15 μW for 30–60 min per sample. Data were recorded using SymPhoTime software 64, version 2.4 (PicoQuant). Data were analyzed with MATLAB-based PAM software (https://pam.readthedocs.io/en/latest/)^119^ using a customized profile optimized for our microscope. Signals from single molecules were observed as bursts of fluorescence. Bursts with more than 50 counts were searched with the dual channel burst search (DCBS) algorithm. Integration time was set to 0.5 ms. Appropriate corrections for direct excitation of the acceptor at the donor excitation wavelength (DE), leakage of the donor in the acceptor channel (Lk), and the instrumental factor (g) were determined experimentally using a mixture of dsDNA models with known FRET efficiency and stoichiometry labeled with Cy3 and Cy5: DE=0.05, Lk=0.08, g=0.85.

### Analytical ultracentrifugation (AUC)

PALB2-DBD samples were dialyzed against buffer (1 M NaCl, 10% glycerol, 1 mM TCEP, 2 mM CHAPS, 25 mM HEPES pH 7.5) consecutively three times for at least 2 h each. For complexes with DNA, protein was first dialyzed against 500 mM NaCl, 10% glycerol, 1 mM TCEP, 2 mM CHAPS, 25 mM HEPES pH 7.5 for at least 6 h, and then mixed with the corresponding DNA substrate while concomitantly reducing NaCl concentration by dilution with buffer without NaCl. Alternatively, DNA was mixed with protein in 0.5 M NaCl and samples were dialyzed against several buffers with a stepwise decreasing concentration of NaCl (350, 250, 100 mM NaCl or 400, 300, 200, 100 mM NaCl and 10% glycerol, 1 mM TCEP, 2 mM CHAPS, 25 mM HEPES pH 7.5).

Experiments were performed using a Beckman Coulter Optima AUC. Samples were loaded into cells and centrifuged at 30,000 rpm for 20 h at 20°C. Absorbance was measured at 280 nm for protein samples and at 550 nm for samples with Cy3-labeled DNA every 4 min for a total of 250 scans. Buffer density and viscosity were calculated using the Sednterp program: 1.069 ρ and 0.01486 η for 1M NaCl buffer, 0.01436 η and 1.04913 ρ for 0.5 M NaCl buffer, and 1.033 ρ and 0.014 η for DNA complexes at 0.1 M NaCl. These values were used for data fitting using the Lamm equation for the continuous c(s) distribution model in Sedfit^120^. Two N-terminal fragments were used (195W and 295W), which consisted of amino acids 1–195 and 1–295, respectively. The last amino acid was mutated to tryptophan to increase absorption at 280 nm. DNA-only samples were analyzed using a spatial specific volume of 0.55. The sequence for the 25mer substrate is 5’Cy3-TGG CGA CGG CAG CGA GGC TCT CTA C; all other substrates are poly(dT).

## Data Availability

All constructs are freely available on request. SAXS scattering data are deposited to SASDBD (https://www.sasbdb.org/) with accession IDs SAS5082 and SAS5083 for high salt and low salt data sets, respectively. The smFRET data in PTU file format (time-correlated single-photon counting data) are available on request.
